# Supplementary material for: Development of a computer-aided design software for the quantitative evaluation of aesthetic damage
Source: PLoS One. 2019 Dec 18;14(12):e0226322. doi: 10.1371/journal.pone.0226322 (PMC6919621; doi:10.1371/journal.pone.0226322)
Supplement: S1 Code site — 1. (ZIP) [file pone.0226322.s001.zip › [EESC_jr][Projeto]Relatorio_07_21/vendors/select2/docs/_includes/examples/themes-templating-responsive-design.html]

# Themes, templating and responsive design

## Theme support

Select2 supports custom themes using the
theme option
so you can style Select2 to match the rest of your application.

These are using the `classic` theme, which matches the old
look of Select2.

{% highlight js linenos %}
$(".js-example-theme-single").select2({
theme: "classic"
});
$(".js-example-theme-multiple").select2({
theme: "classic"
});
{% endhighlight %}

## Templating

Various display options of the Select2 component can be changed:
You can access the `<option>` element
(or `<optgroup>`) and any attributes on those elements
using `.element`.

Templating is primarily controlled by the
`templateResult`
and `templateSelection`
options.

{% highlight js linenos %}
function formatState (state) {
if (!state.id) { return state.text; }
var $state = $(
' ' + state.text + ''
);
return $state;
};
$(".js-example-templating").select2({
templateResult: formatState
});
{% endhighlight %}

## Responsive design - Percent width

Select2's width can be set to a percentage of its parent to support
responsive design. The two Select2 boxes below are styled to 50% and 75%
width respectively.

{% highlight html linenos %}

{% endhighlight %}

Select2 will do its best to resolve the percent width specified via a
css class, but it is not always possible. The best way to ensure that
Select2 is using a percent based width is to inline the
`style` declaration into the tag.
